# Anatomy in Schools

**Published:** 1875-08

**Authors:** 


### ANATOMY IN PUBLIC SCHOOLS.

When we were boys it was next to impossible to acquire a knowledge of the human frame. The science was not taught in the schools, nor was it considered important that anybody except a doctor or a medical student should know the name or the location of a single bone, or artery, or muscle. Farmers knew a good deal more about their cattle than they did about their children or themselves. It took a college education to give one any sort of a knowledge of the complex, yet simple, human machine.

The doctors encouraged this ignorance. It made the science of medicine and surgery more profitable, it elevated its professors on a higher pedestal, and surrounded them with a deeper air of mystery, to keep the human frame as a sealed book to the larger part of the world. Every disease, every organ of the body, and every remedy must be hidden beneath the dead languages.

Things are somewhat better to-day, though the progress has been slow and small. Anatomy, and physiology, and hygiene have not been taught in the public schools in such a manner as to amount to anything, because there have been no teachers competent to expound these sciences, no suitable models for their elucidation, and no provision, pecuniary or otherwise, for instruction in this important direction.

The young should be taught anatomy among their early studies, and they can only be taught it by "object lessons." You may describe a thing to a child a thousand times, and no clear comprehension of it will exist in the mind. The image is not formed. The language which you use conveys no clear idea. But show them the thing itself, or an exact imitation of it in form and color, and its appearance is never forgotten.

The French have devised imitations of the human body which they call "manikins." They give a very fair idea of the parts which they assume to represent. But they are very expensive and they need an expert anatomist to explain them.

It has remained for our own people—who usually manage to improve upon all old ideas—to produce representations of the human frame in *papier mache* and other plastic substances, at small cost and adequate in themselves to convey to the young or the more advanced students, a reasonable knowledge of thehuman frame, Mr. Wilson McDonald, a famous American sculptor, has for twelve years past, employed the little leisure which he could steal from an engrossing profession, to devise a series of anatomical models, which should be perfect as far as they go, reasonable in cost, durable in construction, ample for the leading purposes of the important study which they are intended to facilitate, and small in cost. The first series consists of the Human Skeleton complete, made of a composition exactly resembling bone. Each separate part is labeled with its English or common name and also with its technical name. The skeleton is ingeniously articulated, so that its principal parts may be detached if desired. The price is $20.

The next in the series is a human figure complete,four feet in height,representing the entire external muscular system, and showing the principle bands, tendons and cords.

The different parts are also labeled in English, and the technical name is placed beneath the common name. Every part is painted in oil, in exact imitation of nature, and the structure has a life-like and pleasing effect. The price is $20.

The last object of the initial series is a trunk or body, the size of an average man. It exhibits the entire internal structure—the lungs, heart, liver, stomach, intestines, arteries, veins, &c. Each organ is labeled, technically and in English. It is painted in oil. and in exact imitation of healthy life, and costs $25.

These are all the objects which are as yet offered for sale. Others are, however, in course of preparation—among them a very large human head and neck, a colossal human foot and a colossal human hand, all having the outer covering or skin removed, and showing the muscles, bands, chords, &c.

These models will enable any person of ordinary intelligence to study the bones of the human frame, their conformation, character, position and use; the muscles, their action, location and use; the internal organs, their functions, shape, location and character.

The knowledge which would be secured with slight labor, by the possession of these models, would enable any one to act intelligently in a thousand emergencies in life. The owner would become a much more valuable citizen in a short space of time. The difference between these models and the French Manikin is great. In the French Manikin there are nearly 2,000 objects, and to the student they are but a confused, inexplicable congregation of muscles, ligaments, nerves, arteries, bands and veins. They are covered with the impenetrable veil of a dead language, and all this in one figure, rendering the association of the names and objects impossible. In the American models, the bones are given separately in one figure, the muscles in another, the internal structure in another. The cost of the French Manikin is from $600 to $1,500; amounting to a prohibition of their use in most cases.

The American and Foreign Publication Company, No. 137 Eighth street, New York, are introducing these models to the Superintendents of Schools and to all who desire to secure the most efficient means of culture in the direction of human Anatomy. They announce that they will be glad to correspond with all who feel an interest in this important topic and that they will forward full descriptive circulars to such as apply.

---

A piece of red pepper the size of your finger-nail, put into meat or vegetables when first beginning to cook, will aid greatly in killing the unpleasant odor arising therefrom. Remember this for boiling cabbage, green beans, onions, chickens, mutton, &c.

---

To Assist the Sight.—Persons of defective sight, when threading a needle should hold it over something white, by which the sight will be assisted.

---

If you wish success in life make perseverence your bosom friend, experience your wise counsellor, caution your elder brother, and hope your guardian genius.

---

Energy will do anything that can be done in this world; and no talents, no circumstances, no opportunities, will make a human being a MAN without it.

---

Honest industry is, after all, man's only sure dependence for the double blessing of a contented mind and comfortable livelihood.

